# Neighbor QTL: an interval mapping method for quantitative trait loci underlying plant neighborhood effects

**DOI:** 10.1093/g3journal/jkab017

**Published:** 2021-01-23

**Authors:** Yasuhiro Sato, Kazuya Takeda, Atsushi J Nagano

**Affiliations:** 1 Precursory Research for Embryonic Science and Technology (PRESTO), Japan Science and Technology Agency, Kawaguchi 332-0012, Japan; 2 Research Institute for Food and Agriculture, Ryukoku University, Shiga 520-2194, Japan; 3 Center for Ecological Research, Kyoto University, Shiga 520-2113, Japan; 4 Faculty of Agriculture, Ryukoku University, Shiga 520-2194, Japan

**Keywords:** Ising model, neighbor effects, QTL mapping, plant-insect interaction, R package

## Abstract

Phenotypes of sessile organisms, such as plants, rely not only on their own genotypes but also on those of neighboring individuals. Previously, we incorporated such neighbor effects into a single-marker regression using the Ising model of ferromagnetism. However, little is known regarding how neighbor effects should be incorporated in quantitative trait locus (QTL) mapping. In this study, we propose a new method for interval QTL mapping of neighbor effects, designated “neighbor QTL,” the algorithm of which includes: (1) obtaining conditional self-genotype probabilities with recombination fraction between flanking markers; (2) calculating conditional neighbor genotypic identity using the self-genotype probabilities; and (3) estimating additive and dominance deviations for neighbor effects. Our simulation using F2 and backcross lines showed that the power to detect neighbor effects increased as the effective range decreased. The neighbor QTL was applied to insect herbivory on Col × Kas recombinant inbred lines of *Arabidopsis thaliana*. Consistent with previous results, the pilot experiment detected a self-QTL effect on the herbivory at the *GLABRA1* locus. Regarding neighbor QTL effects on herbivory, we observed a weak QTL on the top of chromosome 4, at which a weak self-bolting QTL was also identified. The neighbor QTL method is available as an R package (https://cran.r-project.org/package=rNeighborQTL), providing a novel tool to investigate neighbor effects in QTL studies.

## Introduction

Sessile organisms, such as land plants, lack the active mobility required to withdraw from neighboring individuals. Field studies have shown that the phenotypes of an individual plants depend not only on their own genotypes but also on those of neighboring plants (Barbosa *et al.* 2009). Such neighbor effects are mediated by direct (*e.g.*, competition and volatile communication) and indirect (*e.g.*, herbivore and pollinator movements) interactions that modulate complex traits throughout a plant life cycle, including growth (Subrahmaniam *et al.* 2018), defense (Schuman *et al.* 2015; Sato 2018; Tamura *et al.* 2020), and reproduction (Underwood *et al.* 2020). Moreover, increasing evidence suggests that plant–plant interactions within a species may increase population biomass and pest resistance (Zeller *et al.* 2012; Wuest and Niklaus 2018; Yang *et al.* 2019). However, it remains unclear how to best analyze the quantitative trait locus (QTL) underlying key traits that lead to plant neighborhood effects. 

QTL mapping is a well-established approach for the analysis of loci responsible for complex traits (Broman *et al.* 2003, 2019; Broman and Sen 2009). Although genome-wide association studies (GWAS) have now been developed, there are several limitations associated with this approach, including the occurrence of false positive signals due to the population structure (Hayes 2013), as well as the omission of rare small-effect variants in a sample population (Korte and Farlow 2013). While recombination events are limited in experimental crosses, QTL mapping can compensate for GWAS disadvantages in population structure and rare variant detection among unrelated individuals. Once GWAS identifies a pair of target accessions, its biparental population is then subject to QTL mapping (Sonah *et al.* 2015; Crowell *et al.* 2016; Han *et al.* 2018). For example, the joint approach using GWAS and QTL mapping has thus far enabled plant researchers to validate loci controlling traits of ecological or agricultural interest in *Arabidopsis thaliana* (Brachi *et al.* 2010), maize (Crowell *et al.* 2016), soya bean (Sonah *et al.* 2015), and peppers (Han *et al.* 2018). Therefore, QTL mapping provides a complementary analysis for GWAS to further dissect complex traits in plant genetics and breeding (Sonah *et al.* 2015; Rishmawi *et al.* 2017; Han *et al.* 2018; Marchadier *et al.* 2019).

Regarding QTL mapping of plant neighborhood effects, Wuest and Niklaus (2018) have recently conducted mixed planting studies with various combinations of near-isogenic lines in *A. thaliana* and uncovered that a single QTL increased the biomass when a pair of lines having different alleles at the QTL marker were cultivated together. Although the first discovery of neighbor QTL is intriguing, it is not yet possible to detect neighbor QTLs in the absence of exhaustive pairwise experiments comprising combinations of many accessions. In this context, our previous study proposed “neighbor GWAS” that screened neighbor effects from random spatial arrangements of multiple genotypes (Sato *et al.* 2021). The core concept of neighbor GWAS was to consider the Ising model of statistical physics as an inverse problem of single-marker regression, thereby estimating the effects of neighbor genotypic identity on a trait. However, QTL mapping of neighbor effects is more complicated than single-marker analysis as QTL studies employ the maximum likelihood method for interval mapping between flanking markers (Haley and Knott 1992; Jansen 1993; Broman and Sen 2009). Such interval mapping requires a stepwise inference from genotype imputation to phenotype prediction. First, conditional genotype probabilities are obtained from the observed marker genotypes and recombination fractions between flanking markers. Second, phenotypes are inferred using the conditional genotype probabilities and marker effects (Haley and Knott 1992). To adopt interval mapping for neighbor effects, it is necessary to define the effects of neighbor genotypic identity on a quantitative trait.

In this study, we developed an interval mapping method for detecting QTLs that led to neighbor effects. The primary aim of the developed method was to detect such neighbor QTLs from spatial variation in phenotypes rather than partialing out the spatial heterogeneity as a nuisance. The proposed method, “neighbor QTL,” was applied to simulated data and recombinant inbred lines (RILs) of *A. thaliana*. Furthermore, the new QTL method was built into an R package.

## Materials and methods

### Model

First, we developed a basic regression model and subsequently defined QTL effects for interval mapping. To combine neighbor effects and a linear model, we focused on a well-known statistical physics model, Ising model (McCoy and Maillard 2012). The Ising model defines the magnetic energy arising from physical interactions among neighboring magnets. By analogy, we regarded an individual as a magnet, genotypes as dipoles, and a trait as energy. Given the observed traits (or energy), we estimated the interaction coefficients of the Ising model to infer neighbor effects.

#### Joint regression for self and neighbor effects

**Figure 1:**
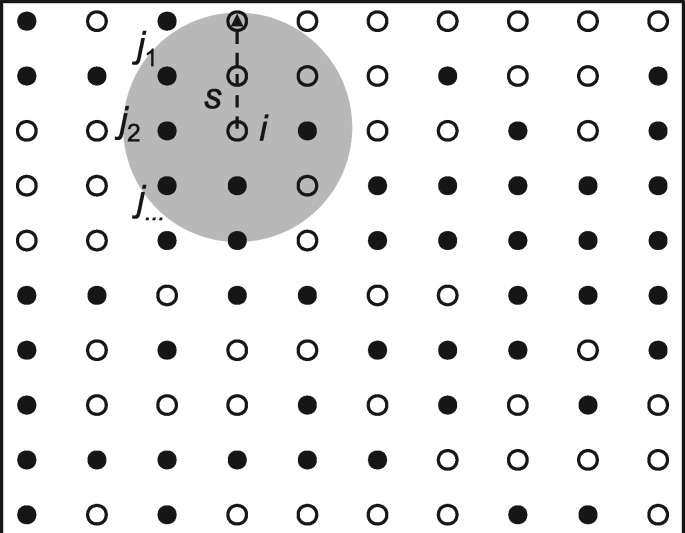
Assumption regarding neighbor effects in a two-dimensional space. A white or black point indicates an individual having AA or BB genotype, respectively. A gray circle represents an effective area of neighbor effects at the spatial distance *s* from the focal individual *i*. Neighbor effects then occur depending on genotype similarity between the focal individual *i* and the *j*-th neighbors within the spatial distance *s*.

To incorporate neighbor effects into linear regression, we developed a joint model following the single-marker regression of neighbor GWAS (Sato *et al.* 2021). We considered a situation where a number of inbred lines occupied finite sites in a two-dimensional space and assumed that an individual is represented by a magnet, whereby two homozygotes at each marker, AA or BB, correspond to the north or south dipole (Figure 1). We defined *x_i_* or *x_j_* as the genotype at a focal marker for *i*-th focal individual or *j*-th neighbor, respectively, where $x_{i(j)}\in${AA, BB} = {1, −1}. We then used multiple regression to model the effects of self-genotype and neighbor genotypic identity on a trait of *i*-th individual *y_i_* as

(1) $$y_{i}=\beta_{0}+\beta_{1}x_{i}+\frac{\beta_{2}}{L}\sum_{<i,j>}^{L}x_{i}x_{j}^{\left(s\right)}+e_{i},$$

where *β*_0_, *β*_1_, and *β*_2_ indicated intercept, self-genotype effects, and neighbor effects, respectively. The residual for a trait value of the focal individual *i* was denoted as *e_i_*. The neighbor genotypic identity was represented by $\sum_{<i,j>}^{L}x_{i}x_{j}^{\left(s\right)}$, which indicated the sum of products for all combinations between the *i*-th focal individual and the *j*-th neighbor at the *s*-th scale of spatial distance from the focal individual *i* (Figure 1). The total number of neighbors *L* varied in response to the spatial scale *s* to be referred. The coefficient of neighbor effects *β*_2_ was scaled by *L*. If two individuals shared the same genotype at a given locus, the product $x_{i}x_{j}$ became positive, whereas the product $x_{i}x_{j}$ became negative if two individuals had different genotypes. Thus, the effects of neighbor genotypic identity on a trait *y_i_* was dependent on the neighbor effect coefficient *β*_2_ and the number of two genotypes in a neighborhood.

Notably, the multiple regression model Equation (1) was posed as an inverse problem of the Ising model. When summing *y_i_* for all individuals and substituting coefficients as $E=-\beta_{2}/L,\;H=-\beta_{1}$ and $\epsilon_{I}=\sum \left(y_{i}-\beta_{0}\right)$, Equation (1) could be transformed into the total magnetic energy of a two-dimensional Ising model as $\epsilon_{I}=-E\sum_{<i,j>}^{L}x_{i}x_{j}^{\left(s\right)}-H\sum x_{i}$ (McCoy and Maillard 2012). In such a case, the neighbor effect *β*_2_ and self-genotype effect *β*_1_ could be interpreted as the interaction coefficient *E* and external magnetic force *H*, respectively.

#### QTL effects of neighbor genotypic identity

To convert a linear regression into a QTL model, we defined QTL effects for self-genotypes and neighbor genotypic identity. With heterozygosity incorporated, we redefined *x_i_* and *x_j_* by a marker genotype for an *i*-th focal individual and *j*-th neighbor as *g_i_* and *g_j_*, respectively. Self-QTL effects expected from these genotypes were denoted as $g_{i(j)}\in${AA, AB, BB} = {*a*_1_, *d*_1_, $-a_{1}$}, where *a*_1_ and *d*_1_ indicated additive and dominance deviation for self-QTL effects, respectively. We then assumed that neighbor QTL effects were not prominent until the two alleles interacted at an individual level and accordingly defined different coefficients of the additive and dominance deviation *a*_2_ and *d*_2_ for neighbor QTL effects. The neighbor QTL effects caused by neighbor genotypic identity between the individual *i* and *j* were defined by its product among nine genotype combinations (Table 1). Given the QTL effects of self-genotype and neighbor genotypic identity, we decomposed a trait of *i*-th individual *y_i_* as,

(2) $$y_{i}=\bar{y}+g_{i}+\frac{\sum_{<i,j>}^{L}g_{i}g_{j}^{\left(s\right)}}{L}+e_{i},$$

where $\bar{y}$ and *e_i_* indicate a population mean of traits and a residual for the focal individual *i*, respectively. If QTL effects were completely additive (*i.e.*, $a_{1}=a_{2}=1$ and $d_{1}=d_{2}=0$), the QTL model Equation (2) had the same structure as the linear regression (Equation 1). In such an additive model, the coefficients *β*_1_ and $\pm \sqrt{\beta_{2}}$ represent additive QTL effects. Notably, the $\pm \sqrt{\beta_{2}}$ sign indicates positive or negative effects of sharing the same alleles on a trait.

**Table 1:** QTL effects expected by genotypic identity between the individuals *i* and *j* with AA, AB, or BB genotypes

*g_i_*/*g_j_*	AA	AB	BB
**AA**	$a_{2}^{2}$	$a_{2}d_{2}$	$-a_{2}^{2}$
**AB**	$a_{2}d_{2}$	$d_{2}^{2}$	$-a_{2}d_{2}$
**BB**	$-a_{2}^{2}$	$-a_{2}d_{2}$	$a_{2}^{2}$

The additive and dominance deviation for the neighbor QTL effects is denoted by *a*_2_ and *d*_2_, respectively.

#### Interval mapping for conditional neighbor genotypic identity

Finally, we extended the single-marker QTL model Equation (2) to interval mapping. In particular, we modified the Haley-Knott regression that approximated the maximum likelihood method by a simple regression (Haley and Knott 1992; Broman and Sen 2009). The proposed algorithm consisted of three steps: (1) obtaining conditional self-genotype probabilities, (2) calculating conditional neighbor genotypic identity from the conditional self-genotype probabilities, and (3) regressing trait values on the conditional self-genotype probabilities and neighbor genotypic identity.

The first step to obtain conditional self-genotype probabilities was the same as that of standard QTL mapping. Let $p_{i(j)}$ be the probability for the focal individual *i* or neighbor *j* to have a certain genotype at an interval pseudo-marker. We defined the conditional self-genotype probability for the individual *i* as $p_{i}=$ Pr(*g_i_* = {AA, AB, BB}|**M**) and obtained *p_i_* from the number of observed markers × *n* individuals matrix **M** and its recombination fraction following hidden Markov models (Lander and Green 1987; Broman *et al.* 2003). Based on the products of the conditional self-genotype probabilities, we further calculated the conditional neighbor genotypic identity $\sum_{<i,j>}^{L}p_{i}p_{j}^{\left(s\right)}/L$. We then defined $g_{i}g_{j}$ as the combination of marker genotypes generating neighbor QTL effects; and $p_{i}p_{j}$ as the expected probability for two genotypes to interact. The expected neighbor QTL combination was accordingly designated as $p_{i}p_{j}g_{i}g_{j}$. These probabilities were summed for all possible combinations of the genotypes as $\sum_{v}^{3}\sum_{w}^{3}[(p_{i,v}p_{j,w}^{\left(s\right)})⊗(g_{i,v}g_{j,w}^{\left(s\right)})]$, where the subscript *v* and *w* indicate the three genotype states AA, AB, and BB.

Similar to Haley-Knott regression, we finally estimated the QTL effects *g_i_* and $g_{i}g_{j}$ by regressing the trait values *y_i_* on *p_i_* and $\sum_{<i,j>}^{L}p_{i}p_{j}^{\left(s\right)}/L$, respectively. The additive and dominance deviations for the self-QTL effects *a*_1_ and *d*_1_ were considered as average differences in trait values among AA, AB, or BB genotypes, such that $a_{1}=({\bar{y}}_{\text{AA}}-{\bar{y}}_{\text{BB}})/2$ and $d_{1}={\bar{y}}_{\text{AB}}-({\bar{y}}_{\text{AA}}+{\bar{y}}_{\text{BB}})/2$ (Broman and Sen 2009). In such a case, the regression coefficient *β*_1_ gave $2{\hat{a}}_{1}$ when -1, 0, and 1 dummy groups were assigned for the AA, AB, and BB genotypes, respectively, or gave ${\hat{d}}_{1}$ when 0, 1, and 0 were assigned for the AA, AB, and BB genotypes, respectively (Broman *et al.* 2003).

**Figure 2:**
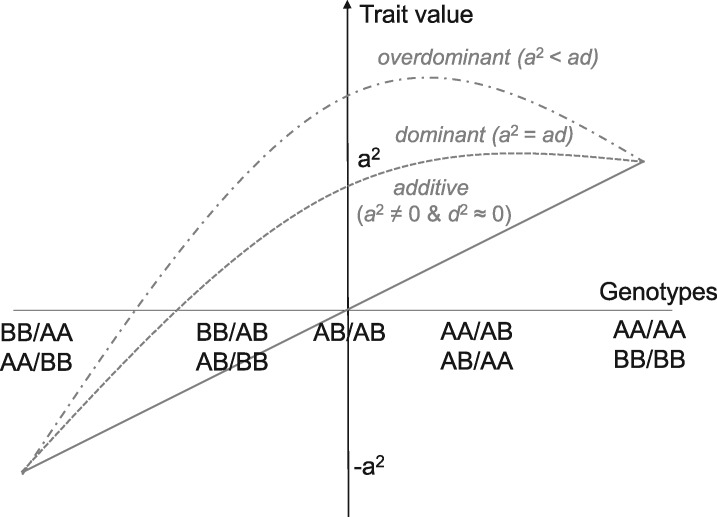
A scheme explaining approximation of neighbor QTL effects by quadratic regression. Trait values *y_i_* are regressed on nine possible combinations of genotype identity between a focal individual *i* and its neighbor *j* (Table 1). The additive or dominance deviation *a* or *d* is represented by the linear or quadratic term, respectively. A negative linear coefficient indicates that sharing of the same alleles (*i.e.*, AA/AA or BB/BB combinations) had negative effects on traits.

For neighbor QTL effects, the additive and dominance deviation *a*_2_ and *d*_2_ were also considered as the average differences in trait values among the nine possible combinations (Table 1) as $a_{2}=[({\bar{y}}_{\text{AA}/\text{AA}}+{\bar{y}}_{\text{BB}/\text{BB}})-({\bar{y}}_{\text{AA}/\text{BB}}+{\bar{y}}_{\text{BB}/\text{AA}})]/4$ and $d_{2}={\bar{y}}_{\text{AB}/\text{AB}}-({\bar{y}}_{\text{AB}/\text{AA}}+{\bar{y}}_{\text{AB}/\text{BB}}+{\bar{y}}_{\text{AA}/\text{AB}}+{\bar{y}}_{\text{BB}/\text{AB}})/4-({\bar{y}}_{\text{AA}/\text{AA}}+{\bar{y}}_{\text{BB}/\text{BB}}+{\bar{y}}_{\text{AA}/\text{BB}}+{\bar{y}}_{\text{BB}/\text{AA}})/4$. In this case, trait values *y_i_* could be fitted by a quadratic regression on the group of nine genotype combinations (Figure 2). Suppose that $y_{i}=\beta_{0}+\beta_{1}p_{i}+\beta_{2}(\sum_{<i,j>}^{L}p_{i}p_{j}^{\left(s\right)}/L)+\beta_{3}^{2}{\left(\sum_{<i,j>}^{L}p_{i}p_{j}^{\left(s\right)}/L\right)}^{2}$ represents such a quadratic regression, where the linear or quadratic coefficient *β*_2_ or $\beta_{3}^{2}$ provides estimates for the additive or dominance deviation $\pm 2{\hat{a}}_{2}^{2}$ or ${\hat{d}}_{2}^{2}$, respectively. Practically, we could estimate *a*_2_ and *d*_2_ by the quadratic regression of the trait values *y_i_* on the conditional neighbor genotypic identity $\sum_{<i,j>}^{L}p_{i}p_{j}^{\left(s\right)}/L$, with nine genotype combinations encoded as AA/AA, BB/BB, AA/AB, AB/AA, AB/AB, AB/BB, BB/AB, AA/BB, BB/AA = {1, 1, 0.25, 0.25, 0.0, −0.25, −0.25, −1, −1}. The estimated sign of $\pm a_{2}^{2}$ inferred positive or negative effects of sharing same alleles on a trait.

Based on the linear and quadratic regression, we decomposed a trait *y_i_* into self and neighbor QTL effects. Notably, the self and neighbor QTL effects are inevitably correlated because the self-QTL component *g_i_* appears in the second and third term in Equation (2). Specifically, when the effective scale *s* was large, less spatial variation occurred in the neighbor condition $\sum g_{j}^{\left(s\right)}$, and stronger correlations arose between self and neighbor QTL effects. If *a*_1_ and *a*_2_ are simultaneously estimated by a one-step regression, there is a risk that the neighbor QTL effects *a*_2_ may be overrepresented when fitted to correlated components. In addition, given that this study aimed to test neighbor effects, it was important to avoid type I errors against neighbor QTL effects. Thus, neighbor QTL effects were tested in comparison with the residuals of self-QTL effects. We estimated *a*_1_, *d*_1_, *a*_2_, and *d*_2_ by following the six-step iterations.

1. Estimate *a*_1_ by a linear regression on self-genotype probabilities, with −1, 0, and 1 encoded for the AA, AB, and BB genotypes, respectively.
2. Estimate *d*_1_ by a linear regression on self-genotype probabilities, with 0, 1, and 0 encoded for the AA, AB, and BB genotypes, respectively.
3. Calculate self-QTL effects with ${\hat{a}}_{1}$ and ${\hat{d}}_{1}$.
4. Include the self-QTL effect as a covariate at a focal marker.
5. Estimate $\pm a_{2}$ and *d*_2_ by a quadratic regression on the conditional neighbor genotypic identity, with [−1, 1] dummy groups assigned for nine genotype combinations.
6. Calculate joint QTL effects with ${\hat{a}}_{1},\;{\hat{d}}_{1},\;{\hat{a}}_{2}$ and ${\hat{d}}_{2}$.

Based on ${\hat{a}}_{1},\;{\hat{a}}_{2},\;{\hat{d}}_{1}$ and ${\hat{d}}_{2}$, we inferred $\hat{y_{i}}$ and derived log_e_ -likelihood (LL) using model deviance. LOD score for the self or neighbor QTL effects were designated as LOD_self_ = log_10_ [exp(LL_self_ − LL_null_)] or LOD_nei_ = log_10_ [exp(LL_nei_ − LL_self_)], which could be obtained in steps 3 and 6, respectively.

When there were only two genotypes, the quadratic regression was replaced by a linear regression to estimate the additive neighbor QTL effects. For the case of inbred lines lacking AB heterozygotes, we estimated the additive deviation *a*_2_ by a linear regression of trait values *y_i_* on the conditional neighbor genotypic identity $\sum_{<i,j>}^{L}p_{i}p_{j}^{\left(s\right)}/L$, with the 1 and −1 dummy groups assigned for the AA and BB genotypes, respectively. In the case of backcross lines lacking BB homozygotes, the additive deviation corresponded to the dominance deviation so that $d_{2}=-a_{2}$. The additive deviation *a*_2_ could be estimated by a linear regression with the AA and AB genotypes encoded as -1 and 0, respectively. These two linear models were equivalent as both the inbred and backcross lines had two genotypes with additive effects.

#### Variation partitioning with the QTL model

Prior to the genome scan, we estimated the effective spatial scale *s* by calculating the proportion of phenotypic variation explained (PVE) by neighbor effects. Incorporating two random effects into a linear mixed model, we could partition the phenotypic variation into PVE by polygenic self-effects, polygenic neighbor effects, and residuals (Sato *et al.* 2021). According to previous studies (Henderson *et al.* 1959; Kang *et al.* 2008), the linear mixed model can be expressed as:

(3) y=Xβ+Zu+e ,

where **y** indicates a phenotype vector as $y_{i}\in y$; Xβ indicates fixed effects with a matrix including a unit vector and all covariates **X** and a coefficient vector β; **Zu** indicates random effects with $u_{i}\in u$ and a design matrix **Z**; and **e** indicates residuals where $e_{i}\in e$. The random effects and residuals were further decomposed as Var(**u**) = $\sigma_{1}^{2}K_{1}+\sigma_{2}^{2}K_{2}$ and Var(**e**) = $\sigma_{e}^{2}I$, where the *n *×* n* individual similarity matrix for self-genotype or neighbor identity was scaled by the number of markers *q* as $K_{1}=P_{1}^{T}P_{1}/(q-1)$ or $K_{2}=P_{2}^{T}P_{2}/(q-1)$, respectively. Given that one of two alleles is similar between heterozygotes and homozygotes, here, we defined the additive polygenic effects for self-QTLs as $g_{i}\in${AA, AB, BB} = {−1, 0, 1} and for neighbor QTLs as $g_{i}g_{j}\in${AA/AA, BB/BB, AA/AB, AB/AA, AB/AB, AB/BB, BB/AB, AA/BB, BB/AA} = {1, 1, 0.5, 0.5, 0.0, −0.5, −0.5, −1, −1}. In these cases, the *q *×* n* matrix $P_{1}$ included expected self-genotype values as elements $P_{1}=(\sum_{v}^{3}p_{i}g_{i})$, and $K_{1}$ represented a kinship matrix that was calculated from all the pseudo-markers (Broman *et al.* 2019). Similarly, the *q *×* n* matrix $P_{2}$ included the conditional neighbor genotypic identities as elements $P_{2}=(\sum_{<i,j>}^{L}\sum_{v}^{3}\sum_{w}^{3}[(p_{i,v}p_{j,w}^{\left(s\right)})⊗(g_{i}g_{j}^{\left(s\right)})])$, and $K_{2}$ represented a genome-wide structure of conditional neighbor genotypic identity. Based on the three variance component parameters, we calculated PVE by polygenic self or neighbor effects as PVE_self_ = $\sigma_{1}^{2}/(\sigma_{1}^{2}+\sigma_{2}^{2}+\sigma_{e}^{2})$ or PVE_nei_ = $\sigma_{2}^{2}/(\sigma_{1}^{2}+\sigma_{2}^{2}+\sigma_{e}^{2})$. In addition, a marker heritability that represented additive genetic variance was defined as $h^{2}=\sigma_{1}^{2}/(\sigma_{1}^{2}+\sigma_{e}^{2})$ when $\sigma_{2}^{2}$ and *s* were set at 0 in Equation (3).

Using the linear mixed model (Equation 3), our previous simulations revealed that increasing patterns of PVE_nei_ from *s *=* *0 to a large *s* (Sato *et al.* 2021) could inform the effective spatial scale of neighbor effects. If the effective range was narrow, PVE_nei_ approached a plateau at a small value of *s*. In contrast, PVE_nei_ linearly increased with *s* if the effective range was broad. To generalize these results for a continuous two-dimensional space, here we introduced ΔPVE metric as differences in PVE from *s* to *s *+* *1 such that ΔPVE = PVE_nei,__*s*__+ 1_ - PVE_nei,__*s*__._ Using such differential metrics, we quantified how PVE_nei_ approached to a plateau across *s* as follows:

1. Categorize spatial scales as s∈S based on the percentiles for pairwise Euclidean distance between individuals.
2. Calculate ${\text{PVE}}_{\mathrm{nei}}$ from *s* = 1 to the maximum elements of *S*.
3. Calculate $\Delta {\text{PVE}}_{\mathrm{nei}}$ and determine s=argmaxΔPVE_nei_

The proposed algorithm using a differential PVE is referred to as “ΔPVE method” hereafter.

#### An R package, “rNeighborQTL”

In addition, the neighbor QTL method was built into an R package, “rNeighborQTL.” The rNeighborQTL inherited input objects from the R/qtl package (Broman *et al.* 2003), allowing us to save phenotype and genotype data as common “cross” objects. Because of the stepwise testing, the self-QTL effects yielded the same results as standard QTL mapping. For the ΔPVE method, the mixed models Equation (3) were solved using the algorithm of average information restricted maximum likelihood (AI-REML) (Gilmour *et al.* 1995) implemented in the Gaston package (Perdry and Dandine-Roulland 2020). An additional but necessary input file was a spatial map describing the positions of individuals at the *x*- and *y*-axes. The rNeighborQTL package is available via the Comprehensive R Archive Network (CRAN) at https://cran.r-project.org/package=rNeighborQTL.

The rNeighborQTL package included several options to analyze a variety of QTL data. Instead of linear (mixed) models (Equations 1 and 3), logistic (mixed) models could also be selected to handle a binary phenotype (Chen *et al.* 2016; Faraway 2016). Because the logistic mixed model did not provide ${\hat{\sigma }}_{e}^{2}$ (Chen *et al.* 2016; Perdry and Dandine-Roulland 2020), PVE_nei_ was substituted by the ratio of phenotypic variation explained (RVE) to polygenic neighbor effects as RVE_nei_$={\hat{\sigma }}_{2}^{2}/{\hat{\sigma }}_{1}^{2}$ when a binary trait was subject to the ΔPVE method. In addition, the neighbor QTL allowed additional covariates when conducting a genome scan. This option enabled composite interval mapping (Jansen 1993) if genetic markers other than a focal locus were considered covariates. When a significant marker was detected by the single-QTL analysis, it was also possible to test two-way interactions, namely epistasis, between the neighbor QTL effects across a genome.

### Simulation

Furthermore, we performed a benchmark test using simulated data on F2 and backcross lines. With a random spatial map generated, we simulated neighbor effects based on “fake.f2” and “fake.bc” autosome genotypes implemented in the R/qtl package (Broman *et al.* 2003). The spatial positions were sampled from a uniform distribution Unif(1, 100) across a continuous two-dimensional space. We estimated *a*_1_ for self-phenotypes of “fake.f2” and “fake.bc” data after the trait values were scaled to have a mean of zero and variance of 1 and assigned $\max{\hat{a}}_{1}$ to a randomly selected marker. In contrast to the major-effect marker, small coefficients, *i.e.*, $10^{-3}\times \max\;{\hat{a}}_{1}$, were assigned to the other markers to simulate polygenic effects. Additive ($a_{2}=\max\;{\hat{a}}_{1}$ and $d_{2}=0.25\times \max\;{\hat{a}}_{1}$), dominant ($a_{2}=d_{2}=\max\;{\hat{a}}_{1}$), and overdominant ($a_{2}=\max\;{\hat{a}}_{1}$ and $d_{2}=1.25\times \max\;{\hat{a}}_{1}$) scenarios were analyzed for the F2 lines, while only additive scenario ($a_{2}=\max{\hat{a}}_{1}$ and $d_{2}=-\max\;{\hat{a}}_{1}$) was applicable for the backcross lines. In total, 30 traits were simulated for true effective distances given at 10-th to 50-th percentiles of pairwise Euclidean distance among individuals. The trait values of simulated neighbor effects were added to the self-phenotypes of “fake.f2” or “fake.bc” data set, with 50% of phenotypic variation being attributable to the neighbor effects. The conditional self-genotype probabilities and conditional neighbor genotypic identity at each marker were standardized to have mean of zero and variance of 1. We then applied the ΔPVE method and a genome scan for the joint traits and calculated LOD_nei_ at s=argmaxΔPVE_nei_ to evaluate the power to detect neighbor effects.

To further examine the model performance, we compared the proportion of PVE by a model including polygenic self-effects alone and a joint model including both the polygenic self and neighbor effects. In addition, we calculated the marker heritability $h^{2}=\sigma_{1}^{2}/(\sigma_{1}^{2}+\sigma_{e}^{2})$ by setting $\sigma_{2}^{2}$ and *s* at 0 in Equation (3). Moreover, the total PVE by the full model was defined by PVE_self_ + PVE_nei_$=(\sigma_{1}^{2}+\sigma_{2}^{2})/(\sigma_{1}^{2}+\sigma_{2}^{2}+\sigma_{e}^{2})$. The PVE unexplained by the heritability but explained by the full model, namely (PVE_self_ + PVE_nei)_$-h^{2}$, could be considered a net contribution of polygenic neighbor effects to phenotypic variation. In addition, to test whether neighbor phenotypes rather than neighbor genotypes explained greater phenotypic variation, we ran the same simulation, with the neighbor genotype $g_{j}^{\left(s\right)}$ replaced by the neighbor phenotype $y_{j}^{\left(s\right)}$ in Equation (3). To assess false positive detection of neighbor genotypic effects due to neighbor phenotypic effects, we also simulated traits involving the neighbor phenotypic effects, the trait values of which were added to the self-phenotypes of “fake.f2” or “fake.bc” data set, with 50% of phenotypic variation attributed to the neighbor phenotypic effects. The simulated traits were then fitted by the neighbor QTL model (*i.e.*, model incorporating neighbor genetic effects).

### Data

To apply the neighbor QTL on real data, we conducted a pilot QTL experiment using the yellow-striped flea beetle *Phyllotreta striolata* and RILs of *A. thaliana* (Supplementary Figure S1A). Adults of flea beetles access host plants by jumping like a “flea.” The adults have a small mouthpart and make small holes on leaves when eating. The number of leaf holes can be used as an indicator of herbivory by flea beetles. These flea beetles are known to prefer glabrous *A. thaliana* to hairy accessions (Sato *et al.* 2019). We selected RILs derived from hairy and glabrous accessions in this study to observe large phenotypic variation in leaf holes.

#### Plants and insects

We used 130 accessions, including parental lines and RILs between Col(*gl1*) and Kas-1 accession (Wilson *et al.* 2001). Col(*gl1*) plants produce no trichomes, while Kas has sparse trichomes on leaves and stems. The RILs are known to vary in the trichome production, disease resistance (Wilson *et al.* 2001), and flowering time (Li *et al.* 2006). We used the marker data based on simple sequence length polymorphism and cleaved amplified polymorphic sequences (Wilson *et al.* 2001). Although SNP markers are available from Li *et al.* (2006), Li *et al.* (2006) analyzed 96 out of the 130 accessions. Furthermore, subsampling of individuals or accessions proved more problematic for neighbor QTL than for standard QTL mapping, as neighbor effects, if any, caused nonindependence of individual data points, and thus, the real influence of neighboring plants on observed traits could not be eliminated from an experiment by subsampling. Therefore, to assess interval mapping capability, we applied the sparse marker data provided by Wilson *et al.* (2001) on the complete set of RIL accessions. The set of RILs was obtained through the Arabidopsis Biological Resource Center (Stock ID, CS84999: https://abrc.osu.edu/).

Flea beetles were maintained under a long-day condition (16:8 h light: dark cycles with a 22${}^{{}^{\circ}}$C constant air temperature) in an environmental chamber (Biotron LH-241PFD-SP, NK system, Osaka, Japan). To establish the experimental population, we collected ca. 200 adults from *Brassica* cultivars grown in the field within Otsu City, Shiga Prefecture, Japan (35${}^{{}^{\circ}}$01′N 135${}^{{}^{\circ}}$51′E) during November 2018 and May 2019. Adults of *P. striolata* consume shoots and especially prefer to young glabrous leaves, whereas larvae consume below-ground tissue of *Brassica* plants; therefore, we reared adults and larvae on leaves and swollen hypocotyls, respectively. Young leaves of Boc choy *Brassica rapa* var. *chinensis* or Chinese cabbage *B. rapa* var. *pekinensis* were supplied for the adults. The larvae were allowed to feed on swollen hypocotyls of the radish *Raphanus sativus* var. *longipinnatus* or the turnip *B. rapa* subsp. *rapa* buried in moisten vermiculite. Adult females laid eggs in the moisten vermiculite, and it took a month (28–32 days) for eggs to become adults.

#### Experimental procedure

To investigate neighbor effects in herbivory, we allowed adult beetles to feed on RIL seedlings grown in a plastic cell tray. Three seeds for each accession were sown on each compartment of the cell tray (13 × 10 cells composed of 20 × 20 mm^2^ compartment) with the accessions randomized. The seeds were acclimated under a constant dark condition with 4${}^{{}^{\circ}}$C for 7 days, and then allowed to germinate under a long-day condition (16:8 h light: dark cycles with a 20${}^{{}^{\circ}}$C constant air temperature). The seedlings were grown under the long-day condition for 24 days, with 2000-fold diluted liquid fertilizer (N:P:K = 6:10:5; Hyponex, Hyponex Japan, Osaka) supplied once. On day 14 post-germination, the seedlings were thinned out to leave one seedling per compartment. Prior to the feeding experiment, we recorded the presence or absence of leaf trichomes and the occurrence of bolting by direct observation and determined the rosette diameter (mm) by analyzing seedling images using the Image J software (Abràmoff *et al.* 2004). The cell tray was enclosed by a white mesh cage (length 29.2 cm × width 41.0 cm × height 27.0 cm: Supplementary Figure S1B). Thereafter, 30 adult beetles were released into the cage and allowed to feed on plants for 72 h. We counted leaf holes as a measure of herbivory for each plant as flea beetles left small holes when they fed on leaves (Supplementary Figure S1C). The final sample size was 126 individuals; out of 130 accessions, 4 accessions (CS84877, CS84873, CS84950, and CS84894) were not germinated, CS84898 lacked genotype data, and CS84958 had two replicates of individuals.

#### Data analysis

We used R version 3.6.0 (R Core Team 2019) for all statistical analyses. A genetic map for the Col × Kas RILs was estimated using the est.map() function in the R/qtl package (Broman *et al.* 2003). Self-genotype probabilities were calculated using the calc.genoprob() function implemented in the R/qtl package (Broman *et al.* 2003). The number of leaf holes was log-transformed and analyzed using linear models. The presence of trichomes and bolting was analyzed using logistic models. When analyzing the number of leaf holes, we incorporated the presence or absence of bolting, the rosette diameter, and the edge (or not) of the cell tray into covariates. The neighbor QTL was performed using the rNeighborQTL package developed in this study. A genome-wide significance level was determined by empirical percentiles of the maximum LOD score among 999 permuted traits. We considered *p *<* *0.1 and *p *<* *0.05 a suggestive and significant level, respectively. In addition, we set an arbitrary threshold at LOD score of 1.5 when discussing the results. To determine whether neighbor phenotypic effects more accurately explained the leaf holes compared to neighbor genotypic effects, we calculated PVE by incorporating the neighbor phenotype $y_{j}^{\left(s\right)}$ instead of neighbor genotype $g_{j}^{\left(s\right)}$ in Equation (3).

### Data availability

R source codes and *Arabidopsis* RIL data set are available in the rNeighborQTL package (https://cran.r-project.org/package=rNeighborQTL). The *Arabidopsis* RIL data include both the marker and phenotype information. Detailed usage of each function and argument is described in the documentation of the rNeighborQTL package. Simulation examples and the analysis of *Arabidopsis* data set are shown in the vignette of the rNeighborQTL package. Photographs of study plants and insects are shown in Supplementary Figure S1. Additional results of the simulation are shown in Supplementary Figure S2. Self-QTL results of *Arabidopsis* RIL data analyzed by the R/qtl package are available in Supplementary Figure S3 as well as in the vignette of the rNeighborQTL package. Additional epistasis analysis of neighbor QTL effects on the leaf holes is shown in Supplementary Figure S4. All the supplementary figures are available at figshare: https://doi.org/10.25387/g3.13395617

## Results and discussion

### Simulation using F2 and backcross lines

**Figure 3:**
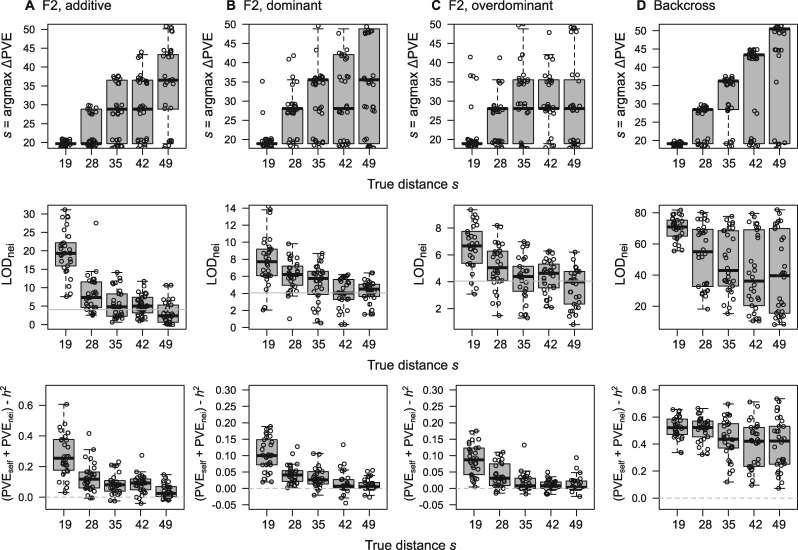
Benchmark test using simulated F2 and backcross data sets. Upper panels show the distance estimated by ΔPVE method. Middle panels show LOD_nei_ of a major-effect marker at the estimated distance. Horizontal solid lines indicate an LOD threshold at *p *=* *0.05 after Bonferroni correction. Lower panels show the net proportion of PVE not by the marker heritability *h*^2^ but by polygenic neighbor effects. Horizontal dashed lines indicate no improvement by the full model that includes both polygenic self and neighbor effects. The *x*-axis corresponds to 10-th to 50-th percentiles of pairwise Euclidean distance, and 30 traits were simulated for each distance class. Boxplots represent median by a center line; upper and lower quartiles by box limits; and 1.5 × interquartile range by whiskers.

We simulated neighbor effects based on “fake.f2” and “fake.bc” data implemented in the R/qtl package (Broman *et al.* 2003). The maximum additive deviation of self-QTL effects, $\max{\hat{a}}_{1}$, was 0.56 and 0.28 for F2 and backcross lines, respectively. These values were assigned for neighbor QTL effects to achieve a similar signal strength between self and neighbor effects, while minor effects were allocated to other loci. Considering the polygenic self and neighbor effects as random effects, we applied the ΔPVE method for simulated traits. The estimated distance given by s=argmaxΔPVE increased as the true distance increased (Figure 3), indicating that the ΔPVE method was effective. Even when neighbor phenotypes were incorporated instead of neighbor genotypes in the model fitting, PVE_nei_ did not increase but decreased in all the four scenarios (mean of the difference in PVE_nei_ = −0.4 for the additive scenario, −0.20 for the dominant scenario, −0.19 for the overdominant scenario, and −0.46 for the backcross lines among 30 iterations from 10-th to 50-th percentiles of the spatial distance class). Incorporating neighbor phenotypes instead of genotypes during the simulation, we applied neighbor QTL to simulated traits involving neighbor phenotypic effects. However, the mean LOD score of a major-effect marker did not exceed 1.60 in any scenario; hence, the neighbor phenotypic effects were unlikely to result in false positive detection of neighbor QTL effects.

When the effective distance of neighbor effects was limited, such short-range neighbor effects were well detected using ΔPVE method and the quadratic approximation (median LOD_nei_ > 4 at the 10-th percentile of pairwise Euclidean distance: Figure 3). Although the power to detect long-range neighbor effects was lowered, the LOD score was still larger than the Bonferroni threshold up to 40-th percentiles of the distance class (median LOD_nei_ > 4: Figure 3). These results indicated that short-range neighbor effects could be detected in any scenario, although it was relatively difficult to detect long-range effects. For backcross lines, both short- and long-range neighbor effects were well detected (median LOD_nei_ > 4 for all *s*: Figure 3D). The backcross lines had two genotypes with the additive deviation alone and were well fitted using linear approximation (Figure 3D), whereas the additive traits for F2 lines were less likely fitted using the quadratic model assuming three genotypes with additive and dominance deviation (Figure 3A). Given the model structure underlying F2 lines, it is plausible that the quadratic term was unnecessary for the additive F2 traits, and it decreased the power to detect neighbor effects.

Linear mixed models including both polygenic self and neighbor effects accounted for a greater proportion of phenotypic variation than the marker heritability that considered polygenic self-effects alone (lower panels in Figure 3). However, the net contribution of polygenic neighbor effects to phenotypic variation, namely (PVE_self_ + PVE_nei)_$-h^{2}$, decreased as the true distance of neighbor effects became larger. In contrast, the marker heritability of simulated traits increased as the true distance of simulated neighbor effects became larger (Supplementary Figure S2). These results were likely due to the correlation between self and neighbor QTL effects, given that the self-QTL component *g_i_* appeared in both the second and third term in Equation (2). Such a correlation became stronger when the effective space broadened and less variation in neighborhood conditions among individuals remained. The loss of PVE by neighbor effects made it difficult to detect long-range neighbor effects as observed in the LOD score simulation (middle panels in Figure 3). To anticipate the underlying correlation structure, we should note that (1) the significance of neighbor QTL effects should be tested using an iterative regression in comparison with the self-QTL model, and (2) PVE_nei_ can be useful for the ΔPVE method but does not indicate the model performance over the self-QTL model.

### Self-QTL effects in Col × Kas RILs

The observed number of leaf holes ranged from 0 to 38 with a median of 4 (Supplementary Figure S1D). The total variation in the number of leaf holes was explained at 5% by the trichome production; 2% by bolting; 10% by the rosette diameter; and 22% by the edge effects (Analysis-of-Variance, F= 9.1, 3.7, 20.7, and 43.4; p= 0.003, 0.06, 10^−4^, and 10^−8^, respectively). With the polygenic self-effects considered a single random effect, a linear mixed model estimated the marker heritability as 5.2% for the leaf holes, though it was not significant (Likelihood ratio test, $\chi_{1}^{2}=1.85,p=0.17$).

**Figure 4:**
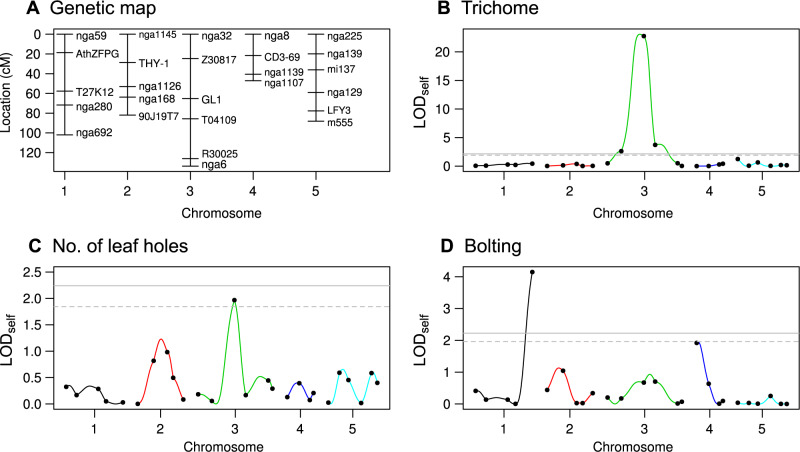
Genetic map and LOD scores for self-QTL effects in Col × Kas RILs. (A) Genetic map showing the locations of 26 markers among the five chromosomes of *A. thaliana*. LOD_self_ score for the trichome production (B), the number of leaf holes (C), bolting (D). Colors correspond to chromosome numbers, and dots indicate observed markers. A solid and dashed horizontal line indicates a significant (*P *<* *0.05) and suggestive (*P *<* *0.1) LOD threshold with 999 permutations, respectively.

To scan self-QTL effects, we conducted standard QTL mapping of the trichome production, the number of leaf holes, and bolting (Figure 4; Table 2). For self-QTL effects on the trichome production, we detected a strong peak near the *GLABRA1* locus (> 20 LOD_self_ score: Figure 4B). Considering the rosette diameter and bolting covariates, we observed a suggestive (0.05<p<0.1) but the largest self-QTL effect on the leaf holes at the *GLABRA1* locus (LOD_self_ = 1.97: Figure 4C). For the bolting, we observed the largest significant peak on the bottom of chromosome 1 (> 4 LOD_self)_, and the second largest and suggestive (0.05<p<0.1) peak on the top of chromosome 4 (LOD_self_ = 1.92: Figure 4D). The self-QTL effects were also analyzed using the R/qtl package. Neighbor QTL yielded the same results regarding the self-QTL effects as the Haley-Knott regression implemented in the scanone() function (Supplementary Figure S3).

**Table 2:** Estimated QTL effects in Col × Kas RILs of *A. thaliana*

Trait	Marker	Chr	Position (cM)	$2a_{1}$	LOD_self_	$2a_{2}^{2}$	LOD_nei_	Distance
**Trichome**	GL1	3	65.24	**−2.83**	**22.8**	3.92	0.31	7.81
**Holes**	GL1	3	65.24	**0.21**	**1.97**	−0.43	0.01	7.81
	nga8	4	0	−0.07	0.13	**2.43**	**1.68**	7.81
**Bolting**	nga692	1	102.0	**−1.04**	**4.15**	**−**1.95	0.66	3.16
	nga8	4	0	**1.05**	**1.92**	1.54	0.15	3.16

Markers with any >1.5 LOD scores (highlighted by bold letters) are shown. Additive effects 2*a*_1_ indicate the effect size when Kas alleles are replaced by two Col alleles, while $2a_{2}^{2}$ indicates the effect size of identical homozygotes over different ones. The sign of $2a_{2}^{2}$ indicates positive or negative effects of sharing same alleles on a trait. The LOD_nei_ score is shown on the spatial distance at which ΔPVE peaks.

Several studies reported the same QTLs or a particular gene function for the self-effects on trichomes, defense, and flowering. Remarkably, *GLABRA1* gene on chromosome 3 is known to encode a myb transcription factor regulating leaf trichome developments (Ishida *et al.* 2008). Our previous study showed that *gl1* mutants were more likely attacked by the flea beetles than hairy wild-types in *A. thaliana* (Sato *et al.* 2019). Other studies on *Brassica* cultivars also documented that leaf trichomes deterred herbivory by flea beetles (Soroka *et al.* 2011; Alahakoon *et al.* 2016). The present finding of the self-QTL effects agrees with the previous evidence for roles of plant trichomes in resistance to flea beetles. Furthermore, two self-bolting QTLs on chromosomes 1 and 4 were located near flowering time QTLs in Col × Kas RILs (Li *et al.* 2006). Thus, our pilot experiment supports previous evidence for the loci responsible for flowering, trichome production, and anti-herbivore defense.

### Neighbor QTL effects in Col × Kas RILs

**Figure 5:**
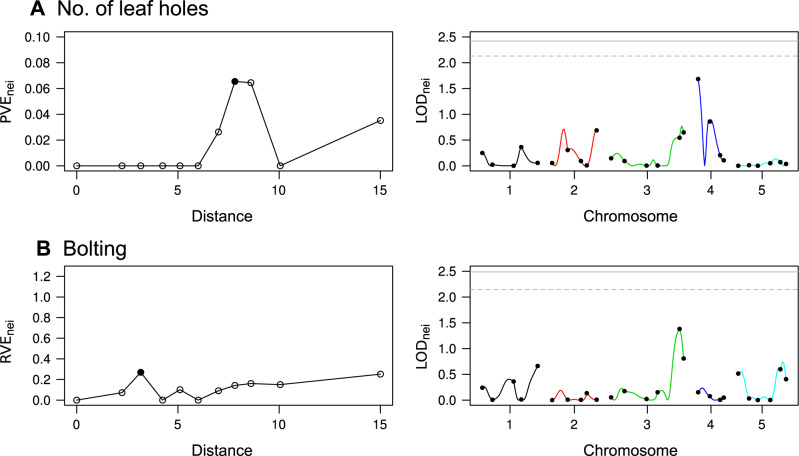
PVE and LOD score attributed to neighbor effects on the number of leaf holes (A) or the presence of bolting (B) in Col × Kas RILs. Left: Proportion, or ratio, of PVE by polygenic neighbor effects (PVE_nei_ or RVE_nei)_ plotted against the pairwise distance among individuals. A closed point indicates the distance at which ΔPVE peaked. Right: LOD_nei_ score for neighbor QTL effects at the distance at which ΔPVE peaked. Colors correspond to chromosome numbers, and dots indicate observed markers. A solid and dashed horizontal line indicates a significant (*P *<* *0.05) and suggestive (*P *<* *0.1) LOD threshold with 999 permutations, respectively.

To estimate the effective distance of neighbor effects, we applied ΔPVE method with every 10-th percentile categories for pairwise Euclidean distance (Figure 5). For the number of leaf holes, the ΔPVE_nei_ was peaked at a 7.81 distance scale from a focal individual (Figure 5A), covering almost all the experimental arena from the center plant. The adults of flea beetles jump and access host plants like a flea. Our ΔPVE method likely reflected such moving behaviors of flea beetles, suggesting that the experimental cage used in this pilot study was too small for flea beetles to mediate neighbor effects among plant individuals. At the estimated distance, 6.3% of total variation in leaf holes was attributable to PVE_nei_ (Figure 5A). The sum of PVE_self_ and PVE_nei_ explained 6.5% of the total variation, although its additional 1.3% fraction compared to 5.2% of the marker heritability was not significant (Likelihood ratio test, $\chi_{1}^{2}=0.23,p=0.62$). Even when neighbor phenotypes were incorporated in place of neighbor genotypes, the PVE for the number of leaf holes did not increase (PVE_nei_ = 0.052, the sum of PVE_self_ and PVE_nei_ = 0.052). Meanwhile, the ΔPVE became the largest at the second nearest scale for the bolting and explained one-third of variation compared to the self-QTL effects (RVE_nei_=0.32 at *s *=* *3.16: Figure 5B), suggesting that the bolting was unlikely to be affected by distant neighbors. For the trichome production, ΔPVE method revealed that the slight variation can be explained using polygenic neighbor effects (RVE_nei_≈ 0 for *s* from 10-th to 60-th percentiles; or models failed to converge for *s* over 70-th percentiles).

A genome scan for neighbor effects was performed using the estimated spatial distance (Figure 5; Table 2). Regarding the neighbor QTL effects on the leaf holes, we observed a weak, but the largest, QTL on the top of chromosome 4 at the nga8 marker (LOD_nei_ = 1.68: Figure 5A;Table 2), which was also the position of the second largest self-bolting QTL. This neighbor QTL had no significant epistasis as shown by <1.1 LOD scores for all the two-way interactions between the nga8 and other markers (Supplementary Figure S4). Neither the neighbor QTL nor *GLABRA1* locus detected above was overlapped with known self-QTLs of powdery mildew resistance (Wilson *et al.* 2001), suggesting independence of the herbivory QTLs on the disease resistance loci. At the second nearest scale for bolting, we found a very weak neighbor QTL at the R30025 marker on the chromosome 3 (LOD_nei_ = 1.38: Figure 5B); however, we did not detect any neighbor QTLs having an LOD score >1.5.

Ecological studies have shown that plant apparency, which defines how easily an individual plant can be identified, drives neighbor effects through visual crypsis against herbivores (Hambäck *et al.* 2000; Castagneyrol *et al.* 2013; Strauss *et al.* 2015). In this study, the neighbor QTL involved in the leaf holes was located near a self-bolting QTL at the top of chromosome 4, suggesting the potential importance of plant apparency in neighbor effects in anti-herbivore defense. In addition, the positive sign of the additive neighbor effects *a*_2_ at that marker indicated that the number of leaf holes decreased when neighbors had different genotypes (Table 2). This implies that the mixture of flowering and vegetative plants may acquire population-wide resistance to flea beetles since the effective distance of neighbor effect was sufficiently large to encompass almost the entire experimental arena. These results led us to hypothesize that the self-QTL underlying plant apparency might facilitate population-wide anti-herbivore defense, called associational resistance (Hambäck *et al.* 2000), through its pleiotropy on neighbor effects.

### Further applicability and limitation

The theoretical advantage of the Ising model lies in its inference of spatial arrangements that optimize total magnetic energy. Once the self and neighbor coefficients are estimated by a marker-based regression, these coefficients may be able to infer which genotype distributions can minimize or maximize the population-sum of trait values (Sato *et al.* 2021). In the context of neighbor QTL, additive effects suggest that positive and negative *a*_2_ favors clustered or mixed patterns for maximizing the sum of trait values, respectively. However, in cases where dominance effects and epistasis are involved, how such a complex genetic basis affects the optimal spatial arrangement remains unexplored. These potential effects of genetic architecture on a population-level outcome of neighbor effects would be of theoretical as well as empirical interest for future studies.

Superior to the previous neighbor GWAS that assumes additive effects alone, the present neighbor QTL offers the flexibility to deal with heterozygosity. While the neighbor QTL analyzes crossed progeny, the neighbor GWAS analyzes unrelated individuals with a population structure incorporated into a random effect of a linear mixed model (Sato *et al.* 2021). Considering the complementary usage of GWAS and QTL mapping in plant genetics (Sonah *et al.* 2015; Crowell *et al.* 2016; Han *et al.* 2018), the neighbor QTL may be useful to analyze crossed progeny of interacting pairs nominated by the neighbor GWAS. However, the use of neighbor QTL is still restricted to autosomes because the sex-dependent inheritance of neighbor effects remains unknown. Standard QTL mapping on sex chromosomes require from one to three degrees of freedom (Broman *et al.* 2006), and thus, its extension to neighbor effects may be more complex than that of self-QTL effects. In addition, the neighbor QTL approximated the maximum likelihood method by a quadratic regression, in which phenotype variance was assumed to be equal among the nine combinations among three QTL genotypes. Our simulations revealed that the quadratic approximation could handle overdominance but was outperformed by linear approximation if additive effects alone governed a trait. We should thus be aware of statistical models behind the neighbor QTL. Practically, both the intercross and the inbred models might be utilized if a sample population is partially inbred.

While this study involved simulations and a laboratory experiment, environmental similarity other than neighbor genotypic identity might also shape spatial patterns of trait values in large-scale cultivation under outdoor conditions. Such environmental autocorrelation matters when genotype distribution and abiotic conditions (*e.g.*, light and soil nutrients) are clustered together in space. Although allele frequencies are unlikely biased in QTL populations, genotypes may be clustered in a large field where complete randomization is hard. When applying the neighbor QTL to field data, joint modeling with a random effect of spatial autocorrelation, such as SpATS (Rodríguez-Álvarez *et al.* 2018), would allow us to distinguish neighbor QTL effects from environmental similarity.

## Conclusion

The present neighbor QTL, together with the previous neighbor GWAS (Sato *et al.* 2021), provides a novel tool to incorporate neighbor effects into quantitative genetics. These methods may provide insights into the genetic architecture underlying neighbor effects, as exemplified by the pilot study of insect herbivory on *A. thaliana*. Once the neighbor GWAS screens candidate accessions, their crossed progeny can be inspected by the neighbor QTL. The line of R packages, “rNeighborQTL” and “rNeighborGWAS,” help investigate neighbor effects using a complementary set of GWAS and QTL data.
